# Analysis of congenital hearing loss after neonatal hearing screening

**DOI:** 10.3389/fped.2023.1153123

**Published:** 2023-05-15

**Authors:** Gill Verstappen, Ina Foulon, Kelsey Van den Houte, Emilie Heuninck, Bart Van Overmeire, Frans Gordts, Vedat Topsakal

**Affiliations:** ^1^ Department of Otorhinolaryngology—Head and Neck Surgery, Universitair Ziekenhuis Brussel, Vrije Universiteit Brussel, Health Campus, Brussels, Belgium; ^2^ Medical Department/Preventive Medicine, Kind en Gezin-Opgroeien, Vlaamse Overheid, Brussels, Belgium

**Keywords:** congenital hearing loss, neonatal hearing screening, sensorineural hearing loss, genetic deafness, congenital CMV infection

## Abstract

**Introduction:**

Neonates undergo neonatal hearing screening to detect congenital hearing loss at an early stage. Once confirmed, it is necessary to perform an etiological workup to start appropriate treatment. The study objective was to assess the different etiologies, risk factors, and hearing results of infants with permanent hearing loss and to evaluate the efficacy and consequences of the different screening devices over the last 21 years.

**Methods:**

We conducted a single-center retrospective cohort analysis for all neonatal hearing screening program referrals and performed an etiological workup in case of confirmed hearing loss. We analyzed the evolution of the etiological protocols based on these results.

**Results:**

The governmental neonatal hearing screening program referred 545 infants to our center. Hearing loss was confirmed in 362 (66.4%) infants and an audiological workup was performed in 458 (84%) cases. 133 (24.4%) infants were diagnosed with permanent hearing loss. Ninety infants (56 bilateral and 34 unilateral) had sensorineural hearing loss, and the degree was predominantly moderate or profound. The most common etiology in bilateral sensorineural hearing loss was a genetic etiology (32.1%), and in unilateral sensorineural hearing loss, an anatomical abnormality (26.5%). Familial history of hearing loss was the most frequently encountered risk factor.

**Conclusion:**

There is a significant number of false positives after the neonatal hearing screening. Permanent hearing loss is found only in a limited number of infants. During the 21 years of this study, we noticed an increase in etiological diagnoses, especially genetic causes, due to more advanced techniques. Genetic causes and anatomical abnormalities are the most common etiology of bilateral and unilateral sensorineural hearing loss, respectively, but a portion remains unknown after extensive examinations.

## 1. Introduction

Congenital hearing loss is a common birth defect that requires early intervention. Therefore, most developed countries have National Neonatal Hearing Screening (NNHS) programs (1). Flanders, the Dutch-speaking part of Belgium, was one of the first regions in the world to implement such screening in 1998 (2). A two-stage screening program using Automated Auditory Brainstem Response (AABR) was implemented for the screening of hearing loss and conducted at home or in well-baby clinics. In the French-speaking part of Belgium, they use a two-stage screening program as well but with an Automated OtoAcoustic Emissions test (AOAE) at the maternity unit (3). The Joint Committee on Infant Hearing recommends screening infants before they turn one month old, then diagnosing an etiology within two months and finally starting treatment for permanent congenital hearing loss before the age of three months (4). Delayed diagnosis of hearing impairment will not only endanger language and speech development, but it will also jeopardize social and emotional development and academic performance later in life (4, 5). Early rehabilitation is therefore of utmost importance to the individual as well as to the society in cost-benefit analyses (6).

“Kind&Gezin”, recently renamed “Opgroeien” is an agency of the Flemish government that contributes to the well-being of children. The governmental agency deals with the development of children concerning health, prevention, morbidity, etc. (7). They implemented a universal neonatal hearing screening in Flanders by use of Automated Auditory Brainstem Response testing (AABR): the Algo Portable device from 1998 until April 2007 and the Algo 3I device from May 2007 to June 2013. Some minor differences between the two devices, including a 4.6 dB difference in stimulus peak level and different stimulus waveforms, resulted in an increased referral rate with the latter device (8). From 2013 until now, a combination of AABR with Automated Auditory Steady-State Responses (ASSR) is used. The Maico® 11MB device combines fast AABR screening with a CE-Chirp stimulus and an Auditory Steady-State Response (ASSR) algorithm. One of the characteristics of this stimulus is that it covers a wide frequency range in the cochlea. The CE-Chirp stimulus provides an optimal auditory brainstem response at a stimulus level of 35 dB nHL, with greater specificity and shorter test duration compared to the Algo® devices (9). Infants are screened before the end of the first month of life. If the test indicates a referral, it means that the measured threshold exceeds the normative value. After at least 48 h the test is repeated and if a “refer” is confirmed, the infant is referred to a specialized reference center. In 2019, 94.2% of infants born in Flanders were tested by “Opgroeien” and 1.74 out of 1,000 tested infants had a congenital hearing loss (10).

Since the introduction of national hearing screening programs, there has also been an evolution in the etiological workup protocols. For example, PCR analyses have been further developed to identify congenital Cytomegalovirus (cCMV) in dried blood spots, comprehensive genetic testing using massively parallel sequencing techniques showed its effectiveness for hearing loss in 2010 and national gene panels were introduced in 2015 (11–13). The frequency of causative genes varies across populations and ethnicities and even geographical regions (14). The diagnostic window has expanded over the years with a trend in reduced acquired congenital hearing loss because of better perinatal care and a relative increase in recognition of congenital genetic hearing loss because of increasing deafness genes that are identified (15). A correct diagnosis for early childhood hearing loss facilitates an appropriate rehabilitation program, predicts disease progression, comforts parents and possibly their guilt feeling, treats coexisting medical problems and provides genetic counseling (14).

The importance of screening is generally accepted and implemented in countries and health care systems that can afford it. On the downside, overscreening or too stringently screening can become a waste of resources and besides the financial loss, it can provoke unnecessary anxiety in parents of allegedly deaf infants (16). Therefore, it is not only important to simply have NNHS programs accessible but equally significant to evaluate their efficacy and correct the screening protocol in due time wherever necessary. Specifically for the Flemish situation, it is constantly monitored, and annual reports are presented to healthcare workers in the field. Our hospital, located in Brussels, is the only institution in the Brussels region that follows the Flemish NNHS program.

Prior to the start of the study, ethical approval was obtained by the ethical committee of University Hospital Brussels with number B.U.N. 143201836386. Here, we study the infants referred to our center after neonatal hearing screening over the last 21 years. We characterized the demographics, etiology, hearing results, risk factors and follow-up rates of the infants from a single tertiary center located in Brussels, perhaps one of the most multicultural cities of Europe. We will assess strengths and shortcomings of the current neonatal hearing screening program and propose recommendations.

## 2. Material and methods

We collected data of all infants that were referred to our center by the governmental agency “Opgroeien” in the context of the neonatal hearing screening program between October 1998 and September 2019. Infants with a hearing impairment that were already in follow-up or even progressive cases with late-onset sensorineural hearing loss were excluded from this study. Admission to a NICU department was also an exclusion criterion since we provide those infants with a hearing test outside the screening program. Once an infant arrives in our center, a full clinical and audiological workup with Auditory Brainstem Responses (ABR), OtoAcoustic Emissions (OAE's) and tympanometry are performed, to determine the type and severity of the congenital hearing loss. The severity of the hearing loss is subdivided into “mild” (31–45 dB nHL), “moderate” (46–70 dB nHL), “severe” (71–90 dB nHL) and “profound” (>91 dB nHL) hearing loss (17). The clinical and full ENT examination actively screens for syndromic features, other external abnormalities, or risk factors for congenital hearing loss. “Opgroeien” registers 11 risk factors, based on those of the Joint Committee on Infant Hearing. These are the following: family history of hearing impairment, craniofacial anomalies, low birth weight, Apgar score 0–4 after one minute or 0–6 after five minutes, artificial respiration for more than five days, parental consanguinity, *in utero* infections (TORCHES), hyperbilirubinemia, bacterial meningitis, ototoxic medication and syndromic abnormality (4). Infants are considered Lost to Follow-Up (LFU) if they did not perform the necessary additional examinations. Normal hearing is defined by hearing thresholds below 40 dB nHL after the first or after a control ABR test.

We initiated the workup, with the necessary additional examinations once we had substantiated the hearing loss. Genetic testing and the possibility of a congenital CMV infection were assessed when dealing with a case of sensorineural hearing loss. Diagnosis of a cCMV infection was made when CMV was detected in urine and/or saliva until three weeks after birth. In most cases, infants presented after this time frame, and the diagnosis of a cCMV infection was made after PCR analysis on dried blood spots (DBS). This is a neonatal screening test that detects rare, non-curative diseases via a few blood drops taken soon after birth, usually via the heel. Until 2015, the etiological workup for sensorineural hearing loss consisted of a “standard test” battery in our center consisting of a cervical vertebral column x-ray, ultrasonography of the kidneys, ECG, and an ophthalmological consultation. The introduction of gene panels has largely replaced or expanded this system in the last couple of years. In the case of bilateral sensorineural hearing loss, we perform genetic testing. In the beginning, only a few genes were screened such as GJB2 and GJB6. This resulted in a low diagnostic window because of the great genetic heterogeneity of hearing loss. Guided by the clinical presentation of sensorineural hearing loss either a gene panel for targeted, syndromic or non-syndromic hearing loss is performed by the laboratories of Center of Medical Genetics (CMG) Antwerp (14).

Most of the infants involved with sensorineural hearing loss underwent radiological imaging, usually an MRI, to evaluate the anatomy of the inner ear and/or retrocochlear structures. In conductive hearing loss cases, such as an ear canal atresia, a CT scan was performed. Sometimes, an additional targeted blood sample or urine analysis was carried out. An Auditory Neuropathy Spectrum Disorder (ANSD) is diagnosed in the absence of ABR thresholds, present otoacoustic emissions and/or cochlear microphonic (CM). Currently, we follow the protocol presented by Liming et al., sometimes supplemented by additional studies based on the clinical presentation (9, 18). If none of the additional tests could identify a cause for the congenital hearing loss, the hearing loss is classified as unknown. After performing the etiological workup, the infant is treated with a multidisciplinary approach, including close collaboration with rehabilitation centers to guide children and their parents with hearing aids, hearing implants, speech therapy, physiotherapy, and psychological help.

## 3. Results

**Figure 1:**
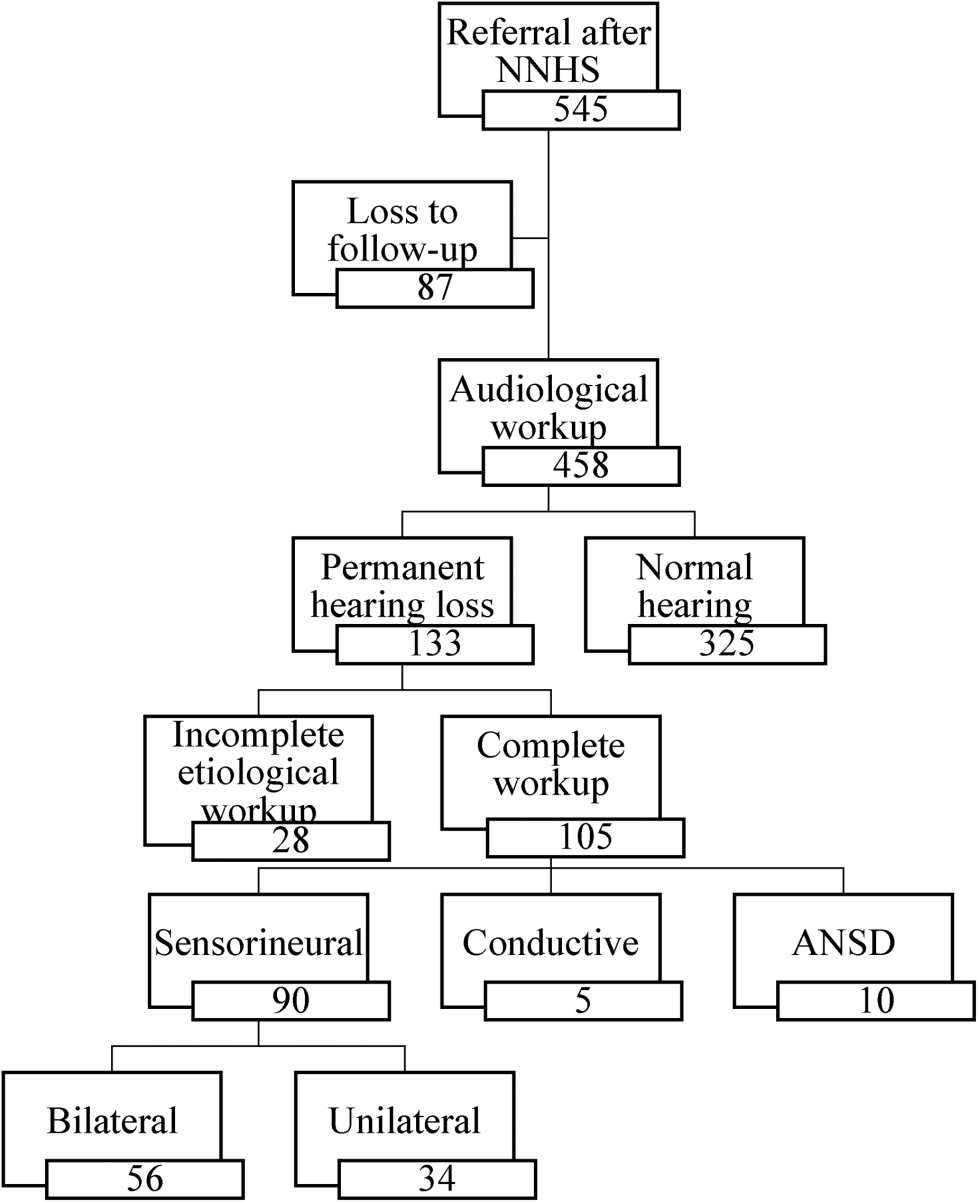
Flow-diagram from screening to diagnosis after audiological workup in our tertiary referral center (NNHS, national neonatal hearing screening; ANSD, auditory neuropathy spectrum disorder).

The Flemish NHS program referred 545 infants (327 boys and 218 girls) for diagnostic analysis for congenital hearing loss. The Algo Portable device referred 90 infants, the Algo 3I device 178 infants and the Maico 11MB device referred 277 infants. A full audiological and an etiological workup was performed on 458 (84%) infants. After etiological workup 325 infants had bilateral normal hearing. This also includes infants with transient hearing loss, for example otitis media with effusion, that disappeared either spontaneously or after placement of tympanic tubes. We diagnosed 133 (24.4%) infants with permanent hearing loss of which 105 received a complete etiological workup (See Figure 1). In 28 infants, parents refused certain additional examinations, resulting in a partial workup. The final diagnosis consisted of 90 infants with sensorineural hearing loss, five infants with conductive hearing loss and ten infants with an Auditory Neuropathy Spectrum Disorder (ANSD). In total 87 (16%) infants were lost to follow-up whereof 54 were diagnosed with otitis media with effusion after the first audiological tests. Additionally, there were 33 infants, of which 23 (69.7%) in the first 10 years and 10 (30.3%) in the last 10 years, with a confirmed hearing loss but without an etiological workup even after actively contacting the parents. In the first half of the study 60.1% of the diagnoses were unknown, in the last half only 40.3%. Furthermore, of the total number of genetic diagnoses, 31.6% were found in the first half and 68.4% in the second half of the study.

**Table 1:** Correlation of the three different screening devices and their number of referrals with the hearing results after the first ABR test.

	Normal hearing		Bilateral hearing loss		Unilateral hearing loss		Total
	*N*	%	*N*	%	*N*	%	*N*
**Algo Portable**							
Bilateral refer	6	16.2	25	67.6	6	16.2	37
Unilateral refer	4	8	15	30	31	62	50
Unknown	3	100	0	0	0	0	3
							90
**Algo 3I**							
Bilateral refer	8	12.7	45	71.4	10	15.9	63
Unilateral refer	36	31.9	26	23	50	44.2	112
Unknown	3	100	0	0	0	0	3
							178
**Maico 11MB**							
Bilateral refer	36	31.3	57	49.6	22	19.1	115
Unilateral refer	83	52.5	17	10.8	58	36.7	158
Unknown	4	100	0	0	0	0	4
							277
**Total**	183	33.6	185	33.9	177	32.5	545

### 3.1. Bilateral sensorineural hearing loss

**Figure 2:**
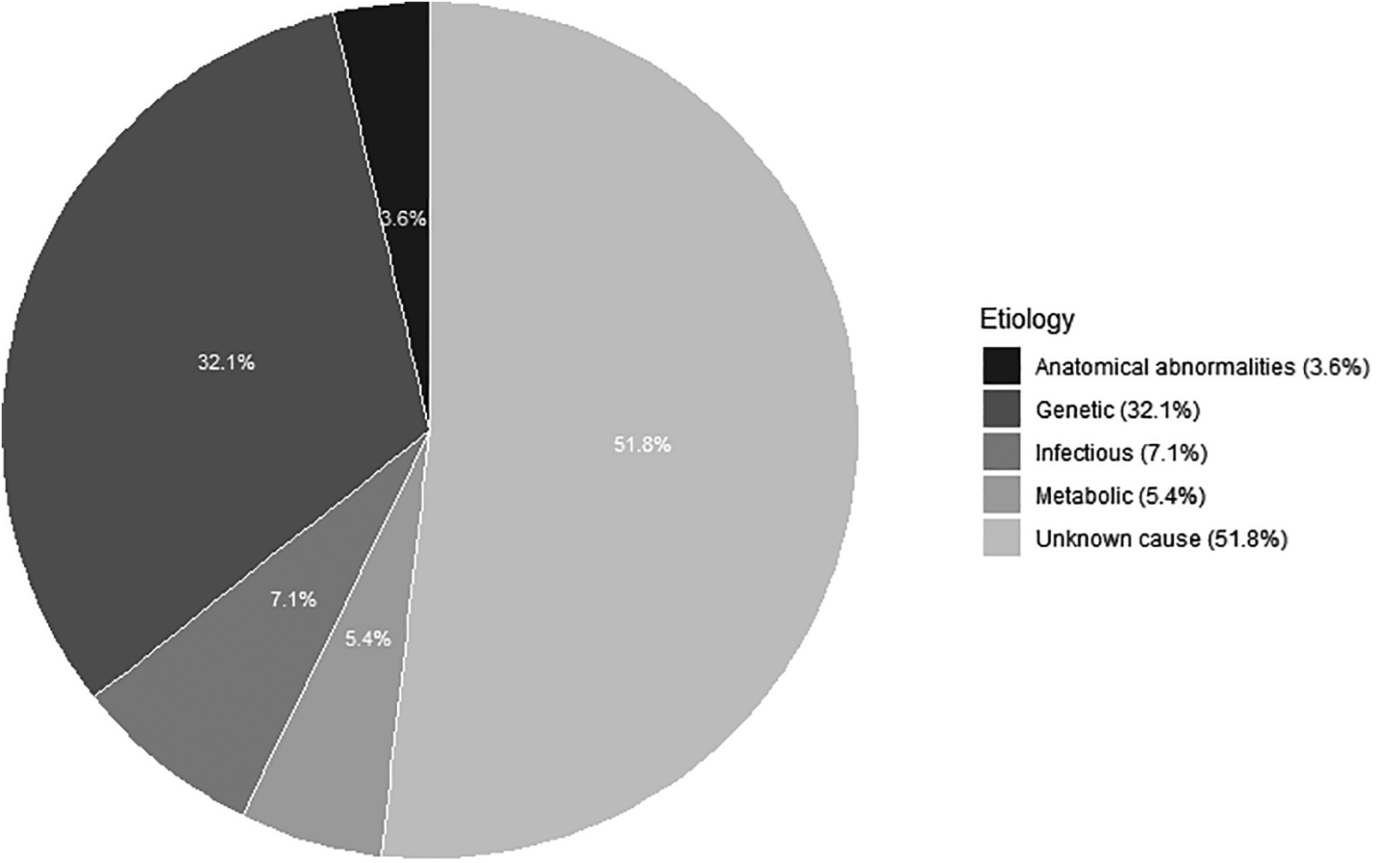
Etiology of bilateral sensorineural hearing loss in 56 children after a complete work-up“ and thus omit ”referred by the Flemish neonatal hearing screening program.

**Figure 3:**
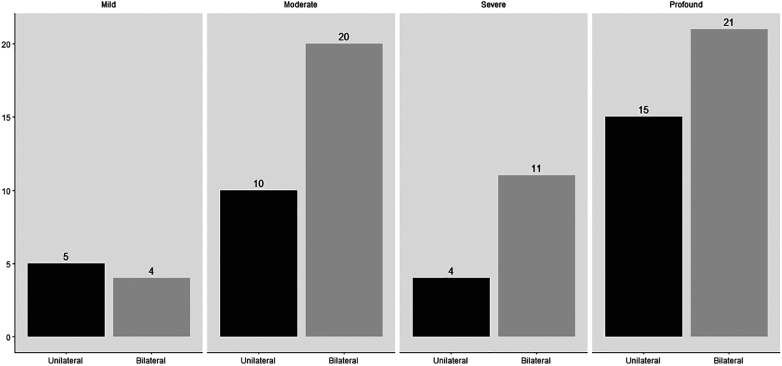
Severity of unilateral or bilateral sensorineural hearing loss of 90 children.

A bilateral sensorineural hearing loss was found in 56 infants (see Figure 2). In 18 (32.1%) infants, a genetic diagnosis could be substantiated. Of these 18 infants, 15 had a non-syndromal cause (83.3%) and three a syndromal cause (16.7%), namely Waardenburg syndrome, KBG syndrome (c.2408_2412del) and Trisomy 21. Of those with a non-syndromic hearing loss, 13 had a connexin mutation of which only one infant had consanguineous parents. The other two infants with a non-syndromic hearing loss had a DFNB16 mutation and a DFNX2 mutation. An infectious cause, always a congenital Cytomegalovirus infection, was found in four (7.1%) of the 56 infants. In three (5.4%) infants, the hearing loss was due to a metabolic cause. Finally, anatomical abnormalities were found in two (3.6%) infants with the aid of an MRI, because of a vestibulocochlear nerve aplasia. In 29 (51.8%) infants with sensorineural hearing loss, the etiology could not be determined after an extensive examination with genetic testing, imaging and CMV analysis and was classified as “unknown”. In those, classified as unknow, 13 had a risk factor for congenital hearing loss, namely consanguinity (2), family history of hearing impairment (9) or both (2). In these infants, a genetic mutation can be suspected. In addition, in one infant, five variants of unclear clinical significance (class III) were also discovered. The diagnostic window for congenital bilateral sensorineural hearing loss over the past 21 years is 48.2%. There were 27 infants with a definite cause of sensorineural hearing loss, of which by 18 a genetic cause was found (66.7%). In Figure 3, the severity of the uni- or bilateral sensorineural hearing loss is presented. The “moderate” and “profound” hearing loss occurred most frequently with respectively 20 and 21 infants.

### 3.2. Unilateral sensorineural hearing loss

A unilateral sensorineural hearing loss was found in 34 infants. The unknown causes and the anatomical abnormalities were the most common etiologies with 20 (58.8%) and nine (26.5%) infants respectively. It includes six with a vestibulocochlear nerve aplasia, one Mondini malformation, one inner ear defect and one enlarged vestibular aqueduct. In addition, a congenital cCMV infection was found in four (11.8%) infants and a genetic cause (trisomy 21) in one infant (3%). No infants with a metabolic pathology were found. Among the unilateral sensorineural hearing losses, “moderate” and “profound” hearing loss were the most common, with ten and fifteen infants respectively (see Figure 3).

### 3.3. Conductive hearing loss

A bilateral conductive hearing loss was seen in five infants in the form of aural atresia (2), ossicle fixation (1) or part of a syndrome (achondroplasia and Hajdu-Cheney syndrome) diagnosed by CT. There were no infants found with a unilateral conductive hearing loss.

### 3.4. Auditory neuropathy/dyssynchrony

An Auditory Neuropathy Spectrum Disorder was diagnosed in ten infants (five unilateral and five bilateral). There was presence of otoacoustic emissions and/or a cochlear microphonic but abnormal or absent auditory brainstem responses.

### 3.5. Risk factors

**Figure 4:**
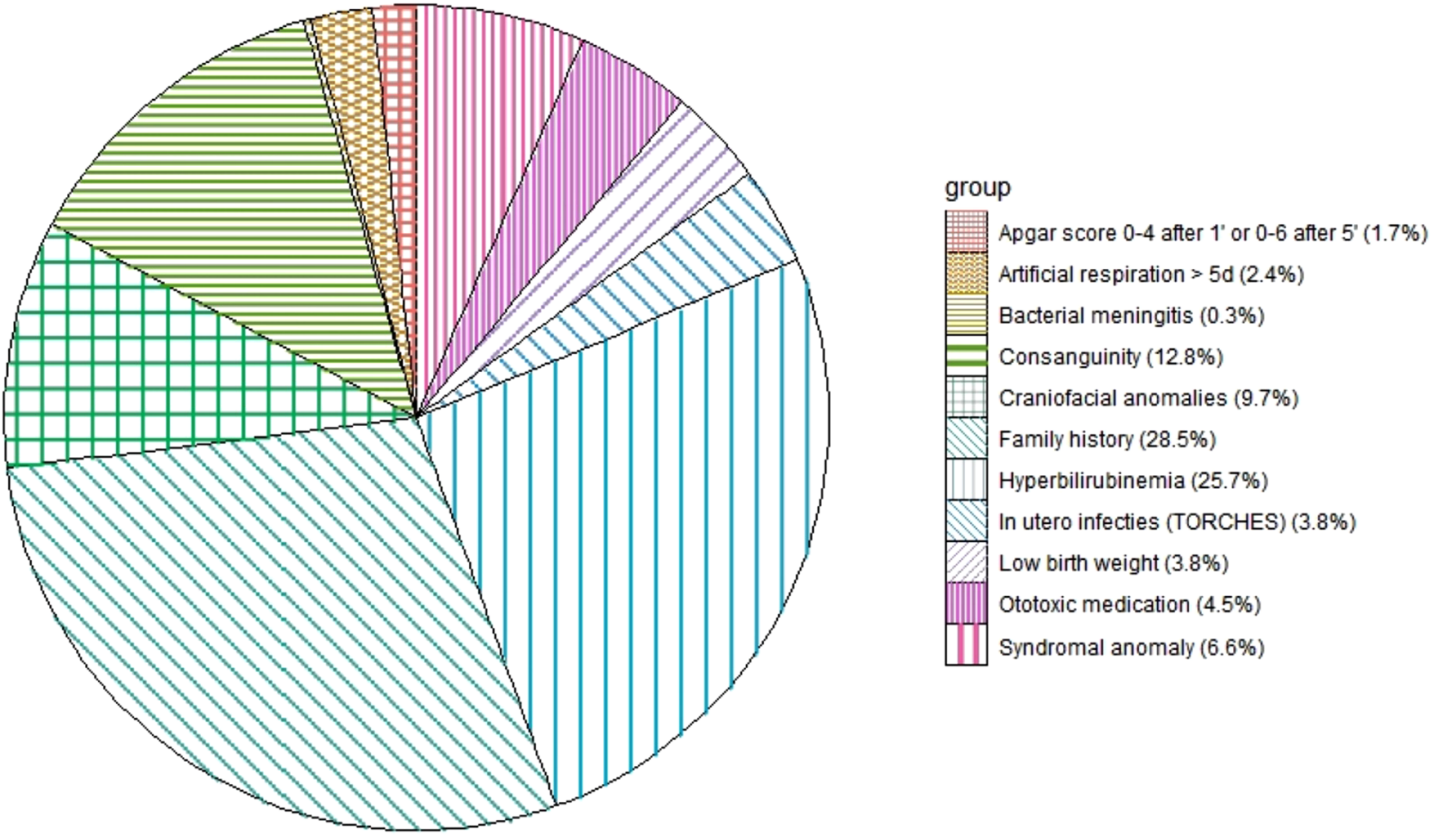
Distribution of the risk factors in 241 children.

At least one of the 11 risk factors was found in 241 (44.2%) of the 545 infants. Of the boys, 45.6% had risk factors in comparison to 42.2% of the girls. A familial history of hearing loss was present in 82 infants or had a 28.5% share in the distribution of risk factors (see Figure 4). Of the infants with a risk factor, 74.3% had a hearing loss after the first ABR test. Of the infants without a risk factor, 60.2% had an initial hearing loss. Of the 133 infants with a permanent hearing loss, 87 infants (65%) had a risk factor for congenital hearing loss and 46 did not (35%).

### 3.6. AABR vs. ABR results

Finally, we correlated the result of the neonatal hearing screening with the audiological result in our reference center (see Table 1). On the one hand, a relative increase in the number of referrals is noted with the introduction of newer screening devices, considering the period of use. On the other hand, we noted that the proportion of normal hearing infants follows this trend in infants who were initially diagnosed with a unilateral or bilateral referral. Only in 362 (66.4%) of the 545 infants, the result of the neonatal hearing screening is confirmed with the first ABR result. In the end, only 133 of the 545 infants (24.4%) are found to have a permanent hearing loss.

## 4. Discussion

The implementation of neonatal hearing screening programs has ensured that infants with congenital hearing loss are detected early, and that rehabilitation can be initiated timely. This is important for language and speech development, social and emotional development, and academic performance later in life (4, 5). The prevalence of permanent bilateral sensorineural congenital hearing loss is 1.33 per 1,000 neonates and has remained stable over the years (19). However, more neonates have been referred to our hospital over the years by changing the screening devices. This is because in Flanders, neonatal hearing screening started with AABR testing and evolved to a combination of AABR and ASSR testing with the latest screening device (8). As a result, in the early years, fewer transient “mild” hearing losses were referred (8, 20). Over the past 21 years, only 133 (24.4%) of the referred infants in our center had a permanent hearing loss. The number of false positives must be monitored because it causes unnecessary anxiety for the parents, costs to society and a decrease in follow-up (16, 21). Another disadvantage of the universal hearing screening program is that progressive, later-acquired or late-onset genetic hearing impairment is not detected (22). This is demonstrated by the fact that the estimated prevalence of permanent bilateral sensorineural hearing loss in primary school children increases to 2.83 per 1,000 and in adolescents to 3.5 per 1,000 (23, 24).

The diagnostic yield after a complete etiological workup for bilateral congenital sensorineural hearing loss in our population over the last 21 years is 48.2%. A genetic cause is found in 32.1% in bilateral sensorineural hearing loss, which is comparable to the study of Van Beeck et al. (25). They found a genetic cause in 27%, an unknown cause in 33% and a suspected genetic cause in 18% (25). Our study was very strict in its classification and didn't make a subdivision for suspected genetic causes. Therefore, there is a high proportion of unknown causes in the bilateral sensorineural hearing loss (51.8%). This is because on the one hand, single gene testing was the norm for many years but insufficient, knowing that the condition is very heterogeneous (26). Gene panels namely, have only relatively recently made their appearance with an increase in the diagnostic window (27). There were also 14 of the 29 infants with an unknown cause where a genetic diagnosis could be expected, considering the familial history of hearing loss, consanguinity, and class III variants. Furthermore, our center is situated in a capital city with a multicultural population. This probably influenced the results, because the number of diagnoses is highly dependent on the age of onset, family history of hearing loss and ethnicity (27). In addition, some mutations are found in our population that currently have unclear clinical significance. These may become a cause of congenital hearing loss in the future, as more data becomes available.

In our population, the genetic causes are due to a non-syndromal cause in 83.3% and to a syndromal cause in only 16.7%. There are fewer syndromic causes found compared to other centers and the available literature (28). Possible causes of this, are that syndromic infants have followed an incomplete etiological protocol or because syndromic infants are referred less often through the neonatal hearing screening program but more often through the NICU, which were not included in the study here.

If imaging is requested at our center, it is almost always an MRI unless a conductive hearing loss is suspected. However, some studies show that in unilateral sensorineural hearing loss, temporal bone abnormalities are found in 29–40% on CT. Only in 10–25% a MRI could show an abnormality (29, 30). We therefore recommend doing both imaging modalities when no genetic or infectious cause can be found. If necessary, this can be done later, e.g., before the placement of a cochlear implant.

In this study, 87 (16%) infants were lost to follow-up, which is similar to other studies but remains an area of concern (31). The transition from screening to intervention is the weakest point in a neonatal hearing screening, where the loss to follow-up and treatment can be as high as 52% of those referred (32). Effective treatment depends largely on the etiology of congenital hearing loss. Some possible factors may contribute to the delay in intervention after diagnosis or LFU: cultural factors, time for reflection by parents, doubt about the benefits of sound amplification devices and doubt about the degree of hearing loss (33). Loss to follow-up can also be due to social, economic and geographic factors (34). After 2008, there were remarkably fewer children with permanent congenital hearing loss in loss to follow-up. This is due to the measures we took after the penultimate position statement appeared in 2007. From then on, we gave a new appointment after every consultation and did not leave this initiative with the parents. If they did not show up at their follow-up appointment, we actively called them and made another appointment. We also took sufficient time for explanations and provided information leaflets, highlighting the importance of follow-up and etiological work-up. Finally, we also ensured closer cooperation with the rehabilitation centers in our neighborhood, exchanging frequent reports on the children's evolution. A possible cause of the residual loss to follow-up could be a consequence of the fact that French-speaking children, after an initial ABR test, are sent to a French-speaking rehabilitation center. From then on, they are followed-up further in the French-speaking pathway and are not in follow-up at our center. Loss to follow-up can have significant consequences for the infant when a congenital hearing loss is not recognized in time in terms of language and speech development (5). Therefore, we need to keep making efforts to try to track down these infants in the future.

In this study could be seen that not only infants with a risk factor have permanent hearing loss but also infants without any risk factors. Therefore, it is still necessary to screen all infants and not only infants with risk factors. In addition, the prevalence of congenital hearing loss is 1.33 per 1,000 in newborns but rises in adolescents to 3.5 per 1,000 (19, 23, 24). This implies that progressive, later-acquired or late-onset genetic hearing impairment is not detected with the neonatal hearing screening program. Congenital Cytomegalovirus infection is a cause in ± 10% of congenital hearing loss in the literature (35). In our study, eight infants (8.9%) had congenital sensorineural hearing loss due to this infection. In childhood, this increases to 15%–20% (35). So, there are also children with a cCMV infection who are not detected. Some children may have an asymptomatic infection that is not picked up in the neonatal hearing screening program because the hearing was initially good and hearing loss only developed later. This is surely a shortcoming in the neonatal hearing screening program. However, Shearer et al. suggested a solution. He suggests an expansion of the neonatal hearing screening program with genetic testing and a cCMV screening complementary to the hearing screening (21). It would, according to recent data, identify more neonates with congenital hearing loss and neonates at risk for hearing loss (36). However, its implementation still faces several challenges, such as gene selection, ethnic bias, current costs and the interpretation of results (21). Still, we may be moving towards such screening in the long run, but further research is needed. Finally, in Belgium there is recently arisen the possibility of doing a genetic carrier screening for couples considering having children in the future. It contains more than 1,000 genes associated with multiple hereditary diseases. The test aims to identify couples that have an increased risk of having a child with a genetic disorder (37). The test also contains genes associated with hearing loss. It could therefore predict prenatally the likelihood of having a child with a hearing loss. Specific genetic tests could then be carried out after birth to confirm the genetic disorder. This way, an etiological diagnosis can be made quickly, and rehabilitation can be started if necessary.

The strengths of this study are the large study population which allowed us to do a thorough etiological and audiological evaluation. Additionally, this study provided a critical review of different screening devices and their impact on the false-positive rate. However, there are some limitations. Firstly, not all available etiological tests were systematically and retrospectively performed in the entire study population. In the early years, additional examinations were progressively requested based on clinical suspicion. In addition, gene panels have only become available in recent years and many genetic diagnoses were probably missed in the early years due to the limited diagnostic methods available at the time. Also, not both imaging modalities (CT and MRI) were requested for all sensorineural hearing losses, which probably meant that some anatomical abnormalities were not found. Another limitation is that the NICU population is not included in this study, since the neonatal hearing screening program is not performed in this population. This could influence the etiological diagnoses and risk factors. Lastly, there is still a significant proportion of infants who are lost to follow-up or did just a partial workup. Unfortunately, we couldn't retrospectively identify the ethnicity of all the infants.

## 5. Conclusion

This study described a significant increase in the number of refers since the change of screening devices with an increase in the number of false-positive referrals. Genetic causes are the most common etiology of bilateral sensorineural hearing loss and anatomical abnormalities of unilateral sensorineural hearing loss, but a large portion remains unknown after extensive examinations. Our data demonstrated an increase in etiological diagnoses over the years, because of the availability of more extensive genetic testing. In the future, attention should be paid to try to further reduce the number of false positives, unknown causes and lost to follow-up. This can be achieved by regularly evaluating the screening protocol, properly involving parents in the workup, and further investigating a possible expansion of the neonatal hearing screening program.

## Data Availability

The original contributions presented in the study are included in the article, further inquiries can be directed to the corresponding author.
